# Crystal structure of {(*E*)-2-[(3,4-di­meth­oxy­phenyl­imino)­meth­yl]phenolato-κ^2^ *N*,*O* ^1^}bis­[2-(pyridin-2-yl)phenyl-κ^2^ *C* ^1^,*N*]iridium(III) di­chloro­methane disolvate

**DOI:** 10.1107/S2056989018009970

**Published:** 2018-07-17

**Authors:** Nirmal K. Shee, Chang Seop Hong, Woo Ram Lee, Hee-Joon Kim

**Affiliations:** a Department of Applied Chemistry, Kumoh National Institute of Technology, Gumi, 39177, Republic of Korea; b Department of Chemistry, Korea University, Seoul 02841, Republic of Korea; c Department of Chemistry, Sejong University, Seoul 05006, Republic of Korea

**Keywords:** crystal structure, cyclo­metalated iridium(III) complex, Schiff base ligand, C_2_N_3_O coordination set

## Abstract

The asymmetric unit of the solvated title complex, [Ir(C_11_H_8_N)_2_(C_15_H_14_NO_3_)]·2CH_2_Cl_2_, consists of two complex mol­ecules together with four di­chloro­methane solvent mol­ecules, one of which is disordered. In each complex mol­ecule, the Ir^III^ ion has a distorted octa­hedral coordination environment defined by two 2-phenyl­pyridine ligands, through two phenyl C and two pyridine N atoms, and by one *N*,*O*-bidentate 2-[(2,4-di­meth­oxy­phenyl­imino)­meth­yl]phenolate anion. The Ir^III^ ions lie almost in the equatorial planes with deviations of 0.0396 (17) and 0.0237 (17) Å, respectively, for the two complex mol­ecules. In both complex mol­ecules, the two 2-phenyl­pyridine ligands are nearly perpendicular to each other [dihedral angles between the least-squares-planes of 89.91 (11) and 85.13 (11)°]. In the crystal, inter­molecular C—H⋯O inter­actions as well as inter­molecular C—H⋯π inter­actions are present, leading to a three-dimensional network structure. One of the four dichlormethane solvent mol­ecules shows disorder over two sets of sites [occupancy ratio 0.79 (2):0.21 (2)].

## Abstract 2

The two Ir^III^ atoms in the title structure have distorted octa­hedral coordination spheres, being *C*,*N*-chelated by two main 2-phenyl­pyridine ligands and *N*,*O*-chelated by one ancillary 2-[(2,4-di­meth­oxy­phenyl­imino)­meth­yl]phenolato ligand.

## Chemical context   

Heteroleptic iridium(III) complexes bearing a coordinating phenyl­pyridine ligand are of great inter­est because of their potential applications in the field of organic light-emitting diodes (OLEDs), as phospho­rescence sensors and in photocatalysis (Evans *et al.*, 2006 ▸; Maity *et al.*, 2015 ▸; Alam *et al.*, 2017 ▸). In particular, cyclo­metalated Ir^III^ complexes with imine-based ancillary ligands exhibit strong aggregation-induced phospho­rescent emission (AIPE) in the solid state (Howarth *et al.*, 2014 ▸; You *et al.*, 2008 ▸). The photophysical properties of these complexes are governed mainly by the coordination environment around the metal ions and the ligand architecture. Hence a small change in the ligand moiety can alter the ground as well as excited states of the metal complexes, making it important to analyze in detail the coordination environment of iridium complexes to understand the origin of phospho­rescence in the solid state (Pal & Singh, 2013 ▸; Goo *et al.*, 2016 ▸).

Here we report the crystal structure of the title compound, [Ir(C_11_H_8_N)_2_(C_15_H_14_NO_3_)]·2CH_2_Cl_2_, a heteroleptic Ir^III^ complex containing a derivative of a salicyl­imine ligand.
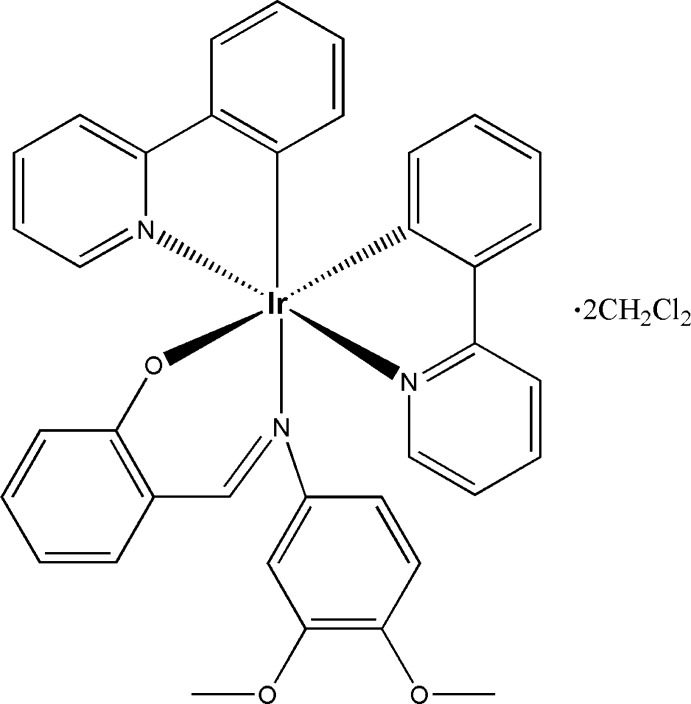


## Structural commentary   

**Figure 1:**
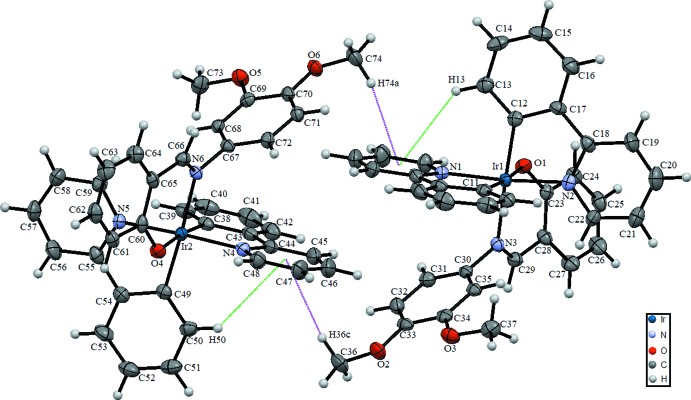
View of the asymmetric unit of the title compound with the atom-numbering scheme. Displacement ellipsoids are drawn at the 50% probability level. Purple and green dashed lines represent intra- and inter­molecular C—H⋯π inter­actions, respectively. Solvent mol­ecules are omitted for clarity.

The asymmetric unit of the title complex consists of two iridium complexes together with four di­chloro­methane solvent mol­ecules. One of the solvent mol­ecules is disordered over two sets of sites. Each complex mol­ecular unit (Fig. 1 ▸) consists of one Ir^III^ ion, two *C,N*-chelating 2-phenyl­pyridine ligands, and one *N,O*-chelating 2-((2,4-di­meth­oxy­phenyl­imino)­meth­yl)phenolate anion. Each Ir^III^ ion adopts a distorted octa­hedral coordination environment defined by two phenyl C, two pyridine N, and one imine and one phenolic O atoms. Selected bond lengths and angles are given in Table 1 ▸ for both complex mol­ecules.

In complex mol­ecule 1 (Ir1), the equatorial plane is defined by atoms O1/N3/C11/C12, the mean deviation from the least-squares plane being 0.044 Å. The Ir1^III^ ion is displaced by 0.0396 (17) Å from the equatorial plane towards the axial N1 atom. The two 2-phenyl­pyridine ligands are nearly planar, with dihedral angles between the aromatic rings of 1.42 (13)° (between rings C6–C11 and N1–C5) and 0.60 (13)° (between rings C12–C17 and N2–C22). The 2-phenyl­pyridine ligands are perpendicular to each other, with a dihedral angle between the least-squares planes of 89.91 (11)°. The coordinating C atoms (C11, C12) are *trans* to the phenolic O1 atom and the imine N3 atom of the anionic ligand, and the two pyridine N atoms (N1 and N2) are also *trans* to each other.

In complex mol­ecule 2 (Ir2), a similar bonding situation is observed, with the phenyl C atoms C38 and C49 *trans* to the O4 and N6 atoms of the 2-[(2,4-di­meth­oxy­phenyl­imino)­meth­yl]phenolate anion. The equatorial plane is formed by atoms O4/C49/C38/N6. The mean deviation from the least-squares plane is 0.055 Å and the Ir2^III^ ion is displaced by 0.0237 (17) Å from the equatorial plane towards the axial N4 atom. The deviation from a perpendicular arrangement of the two 2-phenyl­pyridine ligands is slightly higher than in complex 1 [the dihedral angle between the least-squares planes is 85.13 (11)°], likewise the deviation from planarity with dihedral angles of 1.69 (13)° (between rings C49–C54 and N5–C59) and 3.36 (13)° (between rings C38–C43 and N4–C48), respectively.

The configurations in both complexes are stabilised by intra­molecular C—H⋯O inter­actions between the phenolic O1 and O4 atoms as acceptors and the phenyl C1—H1 and C48—H48 groups as donors (Fig. 1 ▸, Table 2 ▸), as well as by intra­molecular C—H⋯π inter­actions between H13 with *Cg*1 and H50 with *Cg*2 (*Cg*1 and *Cg*2 are the centroids of the N1/C1–C5 and N4/C44–C48 rings, respectively).

The Ir—C, Ir—N, and Ir—O bond lengths, as shown in Table 1 ▸, are consistent with values reported in the literature, *e.g*. for {(*E*)-2-[(2,6-diiso­propyl­phenyl­imino)­meth­yl]phen­o­lato-*κ* ^2^ *N*,*O*}bis­(2-phenyl­pyridine-*κ* ^2^ *C*,*N*)iridium(III) (How­arth *et al.*, 2014 ▸), {(*E*)-2-[(phenyl­imino)­meth­yl]phenolato-*κ* ^2^ *N*,*O*}bis[2-(2,4-di­fluoro­phen­yl)pyridine-*κ* ^2^ *C*,*N*]iridium(III) (You *et al.*, 2008 ▸) or {(*E*)-2-[(phenyl­imino)­meth­yl]phenolato-κ^2^ *N,O*}bis­[2-(pyridin-2-yl)phenyl-κ^2^ *C,N*]iridium(III) (Goo *et al.*, 2016 ▸).

## Supra­molecular features   

In the crystal, the mol­ecules are linked by non-classical C—H⋯O hydrogen-bonds as well as C—H⋯π inter­actions (Figs. 1 ▸ and 2 ▸, Table 2 ▸). Inter­molecular C—H⋯O inter­actions are present between aromatic and methyl donor groups (also involving solvent mol­ecules) and phenolic and meth­oxy O atoms. Additional C—H⋯π inter­actions (Table 2 ▸) are present between H74*a* with *Cg*1 and H36*c* with *Cg*2. The crystal packing lacks any π–π inter­actiosn (negligible above 3.8 Å), although the title compound is very similar to a previously reported compound (Goo *et al.*, 2016 ▸) where this packing feature is present.

## Synthesis and crystallization   

The title compound was prepared according to a reported procedure (Goo *et al.*, 2016 ▸), using 2-[(2,4-di­meth­oxy­phenyl­imino)­meth­yl]phenol instead of 2-[(phenyl­imino)­meth­yl]phenol. Single crystals suitable for X-ray diffrection were obtained by direct diffusion of *n*-hexane (5 mL) into a di­chloro­methane (5 mL) solution of the title compound (6 mg; 8.0 × 10^−3^ mmol) at room temperature.

## Refinement   

Crystal data, data collection and structure refinement details are summarized in Table 3 ▸. All H atoms were positioned geometrically and refined using a riding-model approximation: C—H = 0.95 Å for C*sp* ^2^—H and 0.99 Å for methyl­ene C—H with *U*_iso_(H) = 1.2*U*_eq_(C); C—H = 0.98 Å with *U*_iso_(H) = 1.5*U*_eq_(C) for methyl H atoms. One of the four dichloro­methane solvent mol­ecules shows disorder over two sets of sites [occupancy ratio 0.79 (2):0.21 (2)].

## Supplementary Material

Crystal structure: contains datablock(s) I. DOI: 10.1107/S2056989018009970/wm5449sup1.cif

Structure factors: contains datablock(s) I. DOI: 10.1107/S2056989018009970/wm5449Isup2.hkl

CCDC reference: 1846724

Additional supporting information:  crystallographic information; 3D view; checkCIF report

## Figures and Tables

**Figure 2:**
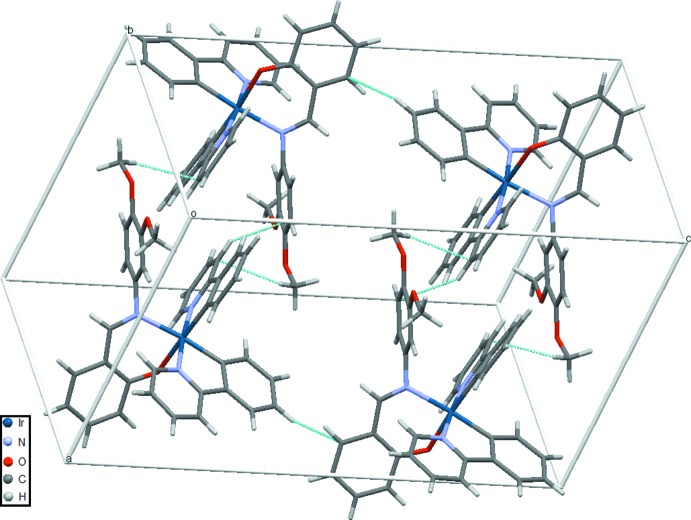
Packing plot of the mol­ecular components in the title compound. Cyan lines represent inter­molecular short contact. Solvent mol­ecules are omitted for clarity.

**Table 1:** Selected bond lengths (Å) and angles (°) for the title complex

Mol­ecule 1 (Ir1)		Mol­ecule 2 (Ir2)	
Ir1—N1	2.028 (3)	Ir2—C38	2.000 (4)
Ir1—C11	1.998 (4)	Ir2—N4	2.030 (3)
Ir1—C12	1.996 (4)	Ir2—C49	2.010 (4)
Ir1—N2	2.036 (3)	Ir2—N5	2.037 (3)
Ir1—O1	2.147 (2)	Ir2—O4	2.151 (2)
Ir1—N3	2.149 (3)	Ir2—N6	2.146 (3)
			
C11—Ir1—N1	80.64 (14)	N4—Ir2—C38	80.63 (14)
C12—Ir1—N1	94.36 (14)	C49—Ir2—C38	86.71 (14)
C12—Ir1—C11	89.53 (14)	C49—Ir2—N4	96.13 (14)
N2—Ir1—N1	174.57 (12)	N5—Ir2—C38	97.04 (14)
N2—Ir1—C11	97.33 (13)	N5—Ir2—N4	176.06 (12)
N2—Ir1—C12	80.55 (14)	N5—Ir2—C49	80.52 (14)
O1—Ir1—N1	94.86 (11)	O4—Ir2—C38	174.17 (13)
O1—Ir1—C11	175.03 (13)	O4—Ir2—N4	95.39 (11)
O1—Ir1—C12	88.70 (12)	O4—Ir2—C49	89.50 (12)
O1—Ir1—N2	86.97 (10)	O4—Ir2—N5	86.69 (11)
N3—Ir1—N1	86.66 (11)	N6—Ir2—C38	97.25 (13)
N3—Ir1—C11	96.53 (13)	N6—Ir2—N4	86.08 (12)
N3—Ir1—C12	173.93 (12)	N6—Ir2—C49	175.75 (12)
N3—Ir1—N2	98.60 (12)	N6—Ir2—N5	97.40 (12)
N3—Ir1—O1	85.25 (10)	N6—Ir2—O4	86.67 (10)

**Table 2:** Hydrogen-bond geometry (Å, °) *Cg*1 and *Cg*2 are the centroids of the N1/C1–C5 and N4/C44–C48 rings, respectively.

*D*—H⋯*A*	*D*—H	H⋯*A*	*D*⋯*A*	*D*—H⋯*A*
C1—H1⋯O1	0.95	2.51	3.112 (5)	121
C13—H13⋯*Cg*1	0.95	3.06	3.83 (4)	139
C74—H74*a*⋯*Cg*1	0.98	3.40	4.12 (5)	132
C48—H48⋯O4	0.95	2.56	3.155 (5)	121
C36—H36*c*⋯*Cg*2	0.98	3.47	4.18 (5)	131
C50—H50⋯*Cg*2	0.95	3.22	3.98 (4)	138
C29—H29⋯O2^i^	0.95	2.60	3.152 (5)	118
C29—H29⋯O3^i^	0.95	2.52	3.463 (5)	172
C58—H58⋯O6^ii^	0.95	2.39	3.330 (5)	170
C75—H75*a*⋯O4^iii^	0.99	2.35	3.310 (7)	165
C77—H77*a*⋯O1^iv^	0.99	2.19	3.172 (7)	172

Symmetry codes: (i) 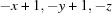; (ii) 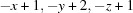; (iii) 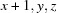; (iv) 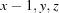.

**Table 3:** Experimental details

Crystal data	
Chemical formula	2[Ir(C_11_H_8_N)_2_(C_15_H_14_NO_3_)]·4CH_2_Cl_2_
*M*_r_	1853.49
Crystal system, space group	Triclinic, *P* 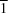
Temperature (K)	130
*a*, *b*, *c* (Å)	12.4000 (5), 14.5371 (6), 21.3502 (8)
α, β, γ (°)	90.112 (1), 106.092 (1), 92.697 (1)
*V* (Å^3^)	3693.2 (3)
*Z*	2
Radiation type	Mo *K*α
μ (mm^−1^)	3.95
Crystal size (mm)	0.25 × 0.14 × 0.05

Data collection	
Diffractometer	Bruker APEXII CCD area detector
Absorption correction	Multi-scan (*SADABS*; Krause *et al.*, 2015 ▸)
*T*_min_, *T*_max_	0.439, 0.827
No. of measured, independent and observed [*I* ≥ 2σ(*I*)] reflections	51940, 17551, 13646
*R*_int_	0.038
(sin θ/λ)_max_ (Å^−1^)	0.668

Refinement	
*R*[*F* ^2^ > 2σ(*F* ^2^)], *wR*(*F* ^2^), *S*	0.032, 0.066, 1.03
No. of reflections	17551
No. of parameters	921
No. of restraints	14
H-atom treatment	All H-atom parameters refined
Δρ_max_, Δρ_min_ (e Å^−3^)	1.25, −1.23

Computer programs: *APEX2* and *SAINT* (Bruker, 2013 ▸), *olex2.solve* and *olex2.refine* (Bourhis *et al.*, 2015 ▸), *OLEX2* (Dolomanov *et al.*, 2009 ▸) and *DIAMOND* (Brandenburg, 2010 ▸).

## supplementary crystallographic information

### Crystal data

**Table:**

2[Ir(C_11_H_8_N)_2_(C_15_H_14_NO_3_)]·4CH_2_Cl_2_	*Z* = 2
*M**_r_* = 1853.49	*F*(000) = 1829.2575
Triclinic, *P*1	*D*_x_ = 1.667 Mg m^−^^3^
*a* = 12.4000 (5) Å	Mo *K*α radiation, λ = 0.71073 Å
*b* = 14.5371 (6) Å	Cell parameters from 9867 reflections
*c* = 21.3502 (8) Å	θ = 2.2–28.4°
α = 90.112 (1)°	µ = 3.95 mm^−^^1^
β = 106.092 (1)°	*T* = 130 K
γ = 92.697 (1)°	Plate, orange
*V* = 3693.2 (3) Å^3^	0.25 × 0.14 × 0.05 mm

### Data collection

**Table:**

Bruker APEXII CCD area detector diffractometer	13646 reflections with *I*≥ 2σ(*I*)
ω scans	*R*_int_ = 0.038
Absorption correction: multi-scan (*SADABS*; Krause *et al.*, 2015)	θ_max_ = 28.3°, θ_min_ = 1.0°
*T*_min_ = 0.439, *T*_max_ = 0.827	*h* = −16→16
51940 measured reflections	*k* = −17→19
17551 independent reflections	*l* = −27→26

### Refinement

**Table:**

Refinement on *F*^2^	14 restraints
Least-squares matrix: full	122 constraints
*R*[*F*^2^ > 2σ(*F*^2^)] = 0.032	All H-atom parameters refined
*wR*(*F*^2^) = 0.066	*w* = 1/[σ^2^(*F*_o_^2^) + (0.0222*P*)^2^ + 3.8204*P*] where *P* = (*F*_o_^2^ + 2*F*_c_^2^)/3
*S* = 1.03	(Δ/σ)_max_ = 0.042
17551 reflections	Δρ_max_ = 1.25 e Å^−^^3^
921 parameters	Δρ_min_ = −1.23 e Å^−^^3^

### Fractional atomic coordinates and isotropic or equivalent isotropic displacement parameters (Å^2^)

**Table:**

	*x*	*y*	*z*	*U*_iso_*/*U*_eq_	Occ. (<1)
Ir1	0.787612 (11)	0.424026 (9)	0.236018 (7)	0.01822 (4)	
N1	0.7554 (2)	0.5519 (2)	0.26199 (14)	0.0212 (7)	
C1	0.8299 (3)	0.6244 (3)	0.2692 (2)	0.0305 (9)	
H1	0.8991 (3)	0.6170 (3)	0.2593 (2)	0.0366 (11)*	
C2	0.8080 (4)	0.7088 (3)	0.2907 (2)	0.0422 (11)	
H2	0.8618 (4)	0.7590 (3)	0.2964 (2)	0.0507 (14)*	
C3	0.7060 (4)	0.7195 (3)	0.3040 (2)	0.0391 (11)	
H3	0.6892 (4)	0.7773 (3)	0.3188 (2)	0.0469 (13)*	
C4	0.6294 (4)	0.6463 (3)	0.2956 (2)	0.0347 (10)	
H4	0.5589 (4)	0.6534 (3)	0.3041 (2)	0.0416 (12)*	
C5	0.6551 (3)	0.5614 (3)	0.27463 (19)	0.0255 (8)	
C6	0.5848 (3)	0.4760 (3)	0.26520 (18)	0.0237 (8)	
C7	0.4773 (3)	0.4692 (3)	0.2740 (2)	0.0329 (10)	
H7	0.4459 (3)	0.5219 (3)	0.2870 (2)	0.0395 (12)*	
C8	0.4164 (3)	0.3854 (3)	0.2636 (2)	0.0368 (11)	
H8	0.3436 (3)	0.3803 (3)	0.2702 (2)	0.0441 (13)*	
C9	0.4616 (3)	0.3096 (3)	0.24380 (19)	0.0319 (10)	
H9	0.4193 (3)	0.2524 (3)	0.23635 (19)	0.0383 (12)*	
C10	0.5682 (3)	0.3158 (3)	0.23460 (18)	0.0250 (8)	
H10	0.5980 (3)	0.2626 (3)	0.22123 (18)	0.0300 (10)*	
C11	0.6326 (3)	0.3987 (3)	0.24456 (17)	0.0218 (8)	
C12	0.8508 (3)	0.3837 (3)	0.32767 (18)	0.0231 (8)	
C13	0.8650 (3)	0.4342 (3)	0.38602 (19)	0.0285 (9)	
H13	0.8377 (3)	0.4944 (3)	0.38418 (19)	0.0342 (11)*	
C14	0.9177 (3)	0.3981 (3)	0.4458 (2)	0.0347 (10)	
H14	0.9262 (3)	0.4339 (3)	0.4843 (2)	0.0417 (12)*	
C15	0.9584 (4)	0.3104 (3)	0.4506 (2)	0.0381 (11)	
H15	0.9953 (4)	0.2865 (3)	0.4920 (2)	0.0457 (13)*	
C16	0.9449 (4)	0.2579 (3)	0.3945 (2)	0.0361 (10)	
H16	0.9718 (4)	0.1976 (3)	0.3974 (2)	0.0433 (12)*	
C17	0.8913 (3)	0.2940 (3)	0.33371 (19)	0.0254 (8)	
C18	0.8737 (3)	0.2435 (3)	0.2723 (2)	0.0263 (9)	
C19	0.9052 (4)	0.1548 (3)	0.2643 (2)	0.0379 (11)	
H19	0.9423 (4)	0.1211 (3)	0.3015 (2)	0.0455 (13)*	
C20	0.8837 (4)	0.1150 (3)	0.2035 (2)	0.0418 (11)	
H20	0.9068 (4)	0.0547 (3)	0.1984 (2)	0.0502 (14)*	
C21	0.8282 (4)	0.1638 (3)	0.1498 (2)	0.0353 (10)	
H21	0.8113 (4)	0.1373 (3)	0.1072 (2)	0.0424 (12)*	
C22	0.7977 (3)	0.2517 (3)	0.15916 (19)	0.0274 (9)	
H22	0.7595 (3)	0.2854 (3)	0.12220 (19)	0.0328 (11)*	
N2	0.8201 (2)	0.2918 (2)	0.21864 (15)	0.0224 (7)	
O1	0.9555 (2)	0.46108 (18)	0.23268 (12)	0.0253 (6)	
C23	0.9886 (3)	0.4459 (2)	0.18083 (18)	0.0213 (8)	
C24	1.1033 (3)	0.4290 (2)	0.18814 (19)	0.0244 (8)	
H24	1.1536 (3)	0.4286 (2)	0.23077 (19)	0.0293 (10)*	
C25	1.1437 (3)	0.4134 (3)	0.1361 (2)	0.0304 (9)	
H25	1.2207 (3)	0.4009 (3)	0.1433 (2)	0.0365 (11)*	
C26	1.0738 (3)	0.4154 (3)	0.0725 (2)	0.0354 (10)	
H26	1.1022 (3)	0.4036 (3)	0.0364 (2)	0.0425 (12)*	
C27	0.9633 (3)	0.4349 (3)	0.0631 (2)	0.0312 (9)	
H27	0.9156 (3)	0.4380 (3)	0.0199 (2)	0.0375 (11)*	
C28	0.9189 (3)	0.4504 (3)	0.11611 (19)	0.0238 (8)	
C29	0.8038 (3)	0.4767 (3)	0.10017 (19)	0.0253 (8)	
H29	0.7739 (3)	0.5015 (3)	0.05814 (19)	0.0304 (10)*	
N3	0.7377 (2)	0.4700 (2)	0.13701 (15)	0.0214 (7)	
C30	0.6300 (3)	0.5092 (3)	0.11168 (17)	0.0215 (8)	
C31	0.6223 (3)	0.6030 (3)	0.10533 (19)	0.0276 (9)	
H31	0.6882 (3)	0.6426 (3)	0.11735 (19)	0.0332 (10)*	
C32	0.5164 (3)	0.6400 (3)	0.08095 (19)	0.0302 (9)	
H32	0.5106 (3)	0.7047 (3)	0.07703 (19)	0.0362 (11)*	
C33	0.4212 (3)	0.5831 (3)	0.06279 (18)	0.0274 (9)	
C34	0.4293 (3)	0.4871 (3)	0.06999 (18)	0.0249 (8)	
C35	0.5330 (3)	0.4508 (3)	0.09482 (17)	0.0217 (8)	
H35	0.5387 (3)	0.3863 (3)	0.10047 (17)	0.0260 (9)*	
O2	0.3141 (2)	0.6130 (2)	0.03783 (14)	0.0360 (7)	
C36	0.3044 (4)	0.7090 (3)	0.0242 (2)	0.0449 (12)	
H36a	0.349 (2)	0.7266 (5)	−0.0057 (12)	0.0673 (18)*	
H36b	0.2254 (5)	0.7213 (5)	0.0041 (14)	0.0673 (18)*	
H36c	0.332 (2)	0.7448 (3)	0.0649 (3)	0.0673 (18)*	
O3	0.3305 (2)	0.43578 (19)	0.05053 (13)	0.0300 (6)	
C37	0.3382 (3)	0.3381 (3)	0.0546 (2)	0.0332 (10)	
H37a	0.3872 (18)	0.3180 (4)	0.0287 (11)	0.0498 (15)*	
H37b	0.370 (2)	0.3211 (3)	0.1002 (3)	0.0498 (15)*	
H37c	0.2632 (4)	0.3083 (3)	0.0376 (12)	0.0498 (15)*	
Ir2	0.231563 (11)	0.938616 (9)	0.272341 (7)	0.01839 (4)	
C38	0.3875 (3)	0.9561 (3)	0.26341 (18)	0.0237 (8)	
C39	0.4595 (3)	1.0350 (3)	0.27746 (19)	0.0297 (9)	
H39	0.4368 (3)	1.0878 (3)	0.29586 (19)	0.0356 (11)*	
C40	0.5638 (3)	1.0374 (3)	0.2649 (2)	0.0381 (11)	
H40	0.6121 (3)	1.0912 (3)	0.2761 (2)	0.0457 (13)*	
C41	0.5984 (3)	0.9630 (3)	0.2366 (2)	0.0422 (12)	
H41	0.6694 (3)	0.9657 (3)	0.2277 (2)	0.0506 (14)*	
C42	0.5286 (3)	0.8846 (3)	0.2214 (2)	0.0375 (11)	
H42	0.5514 (3)	0.8333 (3)	0.2014 (2)	0.0450 (13)*	
C43	0.4243 (3)	0.8800 (3)	0.23504 (19)	0.0270 (9)	
C44	0.3462 (3)	0.7992 (3)	0.22164 (18)	0.0276 (9)	
C45	0.3637 (4)	0.7133 (3)	0.1975 (2)	0.0367 (10)	
H45	0.4327 (4)	0.7031 (3)	0.1881 (2)	0.0441 (12)*	
C46	0.2824 (4)	0.6441 (3)	0.1874 (2)	0.0437 (12)	
H46	0.2938 (4)	0.5863 (3)	0.1701 (2)	0.0524 (14)*	
C47	0.1832 (4)	0.6590 (3)	0.2025 (2)	0.0404 (11)	
H47	0.1259 (4)	0.6114 (3)	0.1963 (2)	0.0484 (13)*	
C48	0.1688 (3)	0.7437 (3)	0.2267 (2)	0.0323 (10)	
H48	0.1007 (3)	0.7538 (3)	0.2373 (2)	0.0388 (11)*	
N4	0.2477 (2)	0.8125 (2)	0.23585 (15)	0.0238 (7)	
C49	0.1800 (3)	1.0004 (3)	0.18569 (18)	0.0233 (8)	
C50	0.1597 (3)	0.9617 (3)	0.12341 (19)	0.0294 (9)	
H50	0.1723 (3)	0.8984 (3)	0.11877 (19)	0.0353 (11)*	
C51	0.1214 (3)	1.0141 (3)	0.0681 (2)	0.0364 (10)	
H51	0.1082 (3)	0.9861 (3)	0.0262 (2)	0.0436 (12)*	
C52	0.1024 (4)	1.1062 (3)	0.0730 (2)	0.0401 (11)	
H52	0.0756 (4)	1.1412 (3)	0.0349 (2)	0.0481 (13)*	
C53	0.1225 (4)	1.1470 (3)	0.1339 (2)	0.0364 (10)	
H53	0.1108 (4)	1.2107 (3)	0.1379 (2)	0.0436 (12)*	
C54	0.1603 (3)	1.0941 (3)	0.18968 (19)	0.0265 (9)	
C55	0.1824 (3)	1.1316 (3)	0.2562 (2)	0.0274 (9)	
C56	0.1672 (4)	1.2216 (3)	0.2727 (2)	0.0395 (11)	
H56	0.1420 (4)	1.2653 (3)	0.2395 (2)	0.0474 (13)*	
C57	0.1888 (4)	1.2474 (3)	0.3373 (2)	0.0389 (11)	
H57	0.1789 (4)	1.3090 (3)	0.3489 (2)	0.0467 (13)*	
C58	0.2248 (3)	1.1832 (3)	0.3851 (2)	0.0343 (10)	
H58	0.2395 (3)	1.1996 (3)	0.4299 (2)	0.0411 (12)*	
C59	0.2389 (3)	1.0951 (3)	0.3665 (2)	0.0272 (9)	
H59	0.2641 (3)	1.0508 (3)	0.3993 (2)	0.0327 (10)*	
N5	0.2183 (2)	1.0690 (2)	0.30361 (15)	0.0217 (7)	
O4	0.05898 (19)	0.91397 (17)	0.27204 (12)	0.0236 (6)	
C60	0.0248 (3)	0.9107 (2)	0.32446 (19)	0.0224 (8)	
C61	−0.0876 (3)	0.9321 (2)	0.3194 (2)	0.0257 (9)	
H61	−0.1340 (3)	0.9504 (2)	0.2785 (2)	0.0308 (10)*	
C62	−0.1314 (3)	0.9272 (3)	0.3716 (2)	0.0297 (9)	
H62	−0.2069 (3)	0.9429 (3)	0.3661 (2)	0.0357 (11)*	
C63	−0.0676 (3)	0.8998 (3)	0.4325 (2)	0.0332 (10)	
H63	−0.0983 (3)	0.8965 (3)	0.4686 (2)	0.0398 (12)*	
C64	0.0417 (3)	0.8775 (3)	0.4387 (2)	0.0283 (9)	
H64	0.0857 (3)	0.8571 (3)	0.4796 (2)	0.0340 (11)*	
C65	0.0906 (3)	0.8838 (2)	0.38691 (19)	0.0216 (8)	
C66	0.2063 (3)	0.8577 (2)	0.40021 (18)	0.0227 (8)	
H66	0.2343 (3)	0.8234 (2)	0.43865 (18)	0.0273 (10)*	
N6	0.2751 (2)	0.8754 (2)	0.36617 (14)	0.0197 (6)	
C67	0.3841 (3)	0.8369 (2)	0.39048 (17)	0.0203 (8)	
C68	0.4788 (3)	0.8960 (2)	0.41121 (17)	0.0200 (8)	
H68	0.4718 (3)	0.9607 (2)	0.40859 (17)	0.0240 (9)*	
C69	0.5831 (3)	0.8604 (2)	0.43559 (18)	0.0211 (8)	
C70	0.5937 (3)	0.7647 (3)	0.43798 (18)	0.0231 (8)	
C71	0.4995 (3)	0.7068 (3)	0.41701 (18)	0.0243 (8)	
H71	0.5065 (3)	0.6420 (3)	0.41879 (18)	0.0291 (10)*	
C72	0.3937 (3)	0.7425 (2)	0.39314 (18)	0.0237 (8)	
H72	0.3288 (3)	0.7023 (2)	0.37881 (18)	0.0284 (10)*	
O5	0.6809 (2)	0.91291 (18)	0.45896 (13)	0.0290 (6)	
C73	0.6730 (3)	1.0102 (3)	0.4541 (2)	0.0318 (10)	
H73a	0.638 (2)	1.0262 (3)	0.4087 (3)	0.0476 (14)*	
H73b	0.6272 (18)	1.0313 (3)	0.4816 (10)	0.0476 (14)*	
H73c	0.7485 (4)	1.0401 (3)	0.4688 (12)	0.0476 (14)*	
O6	0.7018 (2)	0.73717 (18)	0.46183 (13)	0.0286 (6)	
C74	0.7146 (4)	0.6413 (3)	0.4727 (2)	0.0379 (11)	
H74a	0.685 (2)	0.6073 (3)	0.4313 (3)	0.0569 (16)*	
H74b	0.7944 (4)	0.6299 (4)	0.4908 (13)	0.0569 (16)*	
H74c	0.6731 (19)	0.6206 (5)	0.5035 (11)	0.0569 (16)*	
C75	0.8953 (4)	0.8577 (4)	0.1249 (3)	0.0675 (17)	
H75a	0.9343 (4)	0.8666 (4)	0.1718 (3)	0.10 (2)*	
H75b	0.9312 (4)	0.9013 (4)	0.1002 (3)	0.15 (4)*	
Cl1	0.91048 (13)	0.74443 (12)	0.10109 (8)	0.0808 (5)	
Cl2	0.75576 (15)	0.88210 (15)	0.11117 (12)	0.1191 (8)	
C76	0.4050 (4)	0.0541 (3)	0.0954 (3)	0.0478 (12)	
H76a	0.4118 (4)	0.0338 (3)	0.1406 (3)	0.078 (19)*	
H76b	0.3336 (4)	0.0856 (3)	0.0799 (3)	0.11 (2)*	
Cl3	0.51784 (13)	0.13247 (10)	0.09582 (8)	0.0679 (4)	
Cl4	0.40064 (15)	−0.04258 (11)	0.04579 (8)	0.0792 (5)	
C77	0.1228 (3)	0.5166 (3)	0.3705 (2)	0.0361 (10)	
H77a	0.0712 (3)	0.4932 (3)	0.3288 (2)	0.039 (12)*	
H77b	0.1072 (3)	0.4795 (3)	0.4061 (2)	0.057 (15)*	
Cl5	0.26333 (9)	0.50318 (7)	0.36926 (6)	0.0393 (3)	
Cl6	0.09709 (9)	0.63296 (8)	0.38213 (6)	0.0427 (3)	
C78	0.6216 (4)	0.3251 (3)	0.4127 (2)	0.0524 (13)	
H78a	0.5855 (4)	0.3514 (3)	0.3697 (2)	0.0629 (16)*	0.21 (2)
H78b	0.6987 (4)	0.3098 (3)	0.4129 (2)	0.0629 (16)*	0.21 (2)
H78c	0.6104 (4)	0.3378 (3)	0.3658 (2)	0.0629 (16)*	0.79 (2)
H78d	0.7029 (4)	0.3183 (3)	0.4330 (2)	0.0629 (16)*	0.79 (2)
Cl8A	0.6309 (11)	0.4091 (9)	0.4729 (6)	0.047 (4)	0.21 (2)
Cl8B	0.5743 (11)	0.41843 (18)	0.4508 (4)	0.108 (3)	0.79 (2)
Cl7	0.54746 (15)	0.22451 (9)	0.42112 (7)	0.0723 (5)	

### Atomic displacement parameters (Å^2^)

**Table:**

	*U*^11^	*U*^22^	*U*^33^	*U*^12^	*U*^13^	*U*^23^
Ir1	0.01840 (7)	0.01837 (8)	0.01894 (8)	0.00189 (5)	0.00675 (6)	0.00085 (6)
N1	0.0242 (15)	0.0217 (16)	0.0175 (16)	0.0041 (13)	0.0048 (13)	0.0007 (13)
C1	0.031 (2)	0.024 (2)	0.037 (3)	−0.0007 (17)	0.0106 (19)	−0.0019 (18)
C2	0.047 (3)	0.022 (2)	0.056 (3)	0.0007 (19)	0.011 (2)	−0.003 (2)
C3	0.052 (3)	0.026 (2)	0.039 (3)	0.013 (2)	0.010 (2)	−0.0028 (19)
C4	0.039 (2)	0.040 (3)	0.026 (2)	0.017 (2)	0.0085 (19)	−0.0025 (19)
C5	0.0276 (19)	0.028 (2)	0.023 (2)	0.0105 (16)	0.0093 (16)	0.0057 (16)
C6	0.0206 (18)	0.034 (2)	0.018 (2)	0.0039 (16)	0.0070 (15)	0.0046 (16)
C7	0.027 (2)	0.042 (3)	0.032 (2)	0.0144 (18)	0.0112 (18)	0.010 (2)
C8	0.0196 (19)	0.056 (3)	0.039 (3)	0.0042 (19)	0.0143 (18)	0.010 (2)
C9	0.026 (2)	0.038 (2)	0.030 (2)	−0.0064 (18)	0.0056 (18)	0.0085 (19)
C10	0.0251 (19)	0.028 (2)	0.024 (2)	0.0018 (16)	0.0099 (16)	0.0035 (16)
C11	0.0222 (18)	0.028 (2)	0.0167 (19)	0.0025 (15)	0.0066 (15)	0.0035 (15)
C12	0.0204 (18)	0.027 (2)	0.024 (2)	0.0006 (15)	0.0095 (16)	0.0011 (16)
C13	0.028 (2)	0.031 (2)	0.027 (2)	0.0009 (17)	0.0088 (17)	0.0009 (17)
C14	0.041 (2)	0.041 (3)	0.020 (2)	−0.012 (2)	0.0086 (19)	−0.0018 (19)
C15	0.038 (2)	0.044 (3)	0.027 (2)	−0.004 (2)	0.0024 (19)	0.013 (2)
C16	0.044 (2)	0.029 (2)	0.034 (3)	0.0051 (19)	0.007 (2)	0.0089 (19)
C17	0.0257 (19)	0.025 (2)	0.023 (2)	0.0010 (16)	0.0035 (16)	0.0060 (16)
C18	0.0258 (19)	0.0197 (19)	0.032 (2)	0.0008 (15)	0.0060 (17)	0.0038 (17)
C19	0.049 (3)	0.022 (2)	0.041 (3)	0.0091 (19)	0.008 (2)	0.0039 (19)
C20	0.049 (3)	0.021 (2)	0.058 (3)	0.003 (2)	0.020 (2)	−0.002 (2)
C21	0.042 (2)	0.029 (2)	0.039 (3)	−0.0005 (19)	0.019 (2)	−0.0092 (19)
C22	0.029 (2)	0.027 (2)	0.027 (2)	0.0011 (16)	0.0088 (17)	−0.0063 (17)
N2	0.0205 (15)	0.0220 (16)	0.0270 (18)	−0.0008 (12)	0.0108 (13)	−0.0013 (14)
O1	0.0189 (13)	0.0331 (15)	0.0237 (15)	−0.0014 (11)	0.0063 (11)	−0.0043 (12)
C23	0.0232 (18)	0.0164 (18)	0.025 (2)	−0.0020 (14)	0.0078 (16)	0.0004 (15)
C24	0.0208 (18)	0.023 (2)	0.029 (2)	−0.0001 (15)	0.0070 (16)	0.0044 (16)
C25	0.028 (2)	0.029 (2)	0.040 (3)	0.0079 (17)	0.0178 (19)	0.0076 (19)
C26	0.035 (2)	0.045 (3)	0.034 (3)	0.0120 (19)	0.020 (2)	0.011 (2)
C27	0.030 (2)	0.041 (2)	0.027 (2)	0.0071 (18)	0.0142 (18)	0.0082 (19)
C28	0.0233 (18)	0.024 (2)	0.026 (2)	0.0034 (15)	0.0092 (16)	0.0066 (16)
C29	0.0262 (19)	0.028 (2)	0.022 (2)	0.0043 (16)	0.0063 (16)	0.0027 (16)
N3	0.0192 (15)	0.0221 (16)	0.0236 (18)	0.0037 (12)	0.0067 (13)	0.0009 (13)
C30	0.0211 (18)	0.030 (2)	0.0153 (19)	0.0077 (15)	0.0062 (15)	0.0006 (15)
C31	0.027 (2)	0.031 (2)	0.024 (2)	0.0018 (17)	0.0063 (17)	0.0008 (17)
C32	0.036 (2)	0.027 (2)	0.029 (2)	0.0101 (18)	0.0110 (18)	0.0021 (17)
C33	0.0233 (19)	0.044 (2)	0.016 (2)	0.0130 (17)	0.0066 (16)	0.0038 (17)
C34	0.0252 (19)	0.035 (2)	0.017 (2)	0.0047 (16)	0.0097 (16)	0.0016 (16)
C35	0.0221 (18)	0.028 (2)	0.0166 (19)	0.0038 (15)	0.0072 (15)	0.0036 (15)
O2	0.0288 (15)	0.0420 (18)	0.0354 (17)	0.0163 (13)	0.0036 (13)	0.0015 (14)
C36	0.041 (3)	0.048 (3)	0.042 (3)	0.025 (2)	0.001 (2)	0.007 (2)
O3	0.0178 (13)	0.0398 (17)	0.0314 (16)	0.0031 (11)	0.0048 (12)	0.0043 (13)
C37	0.027 (2)	0.039 (2)	0.034 (3)	−0.0031 (18)	0.0095 (18)	−0.0030 (19)
Ir2	0.01743 (7)	0.01797 (8)	0.01981 (8)	0.00181 (5)	0.00509 (6)	0.00010 (6)
C38	0.0217 (18)	0.029 (2)	0.020 (2)	0.0039 (15)	0.0057 (15)	0.0069 (16)
C39	0.0246 (19)	0.036 (2)	0.028 (2)	−0.0015 (17)	0.0071 (17)	0.0077 (18)
C40	0.025 (2)	0.049 (3)	0.036 (3)	−0.0076 (19)	0.0025 (19)	0.014 (2)
C41	0.023 (2)	0.065 (3)	0.043 (3)	0.007 (2)	0.015 (2)	0.017 (2)
C42	0.031 (2)	0.053 (3)	0.032 (2)	0.015 (2)	0.0134 (19)	0.009 (2)
C43	0.0234 (19)	0.036 (2)	0.022 (2)	0.0097 (17)	0.0055 (16)	0.0072 (17)
C44	0.029 (2)	0.034 (2)	0.019 (2)	0.0118 (17)	0.0040 (16)	0.0016 (17)
C45	0.043 (3)	0.039 (3)	0.029 (2)	0.020 (2)	0.008 (2)	−0.0014 (19)
C46	0.059 (3)	0.029 (2)	0.039 (3)	0.015 (2)	0.004 (2)	−0.005 (2)
C47	0.045 (3)	0.024 (2)	0.045 (3)	−0.0012 (19)	0.001 (2)	−0.008 (2)
C48	0.032 (2)	0.026 (2)	0.036 (3)	0.0020 (17)	0.0057 (19)	−0.0025 (18)
N4	0.0247 (16)	0.0226 (17)	0.0227 (18)	0.0027 (13)	0.0040 (13)	0.0021 (13)
C49	0.0184 (17)	0.027 (2)	0.025 (2)	0.0013 (15)	0.0067 (16)	0.0019 (16)
C50	0.0239 (19)	0.038 (2)	0.025 (2)	0.0020 (17)	0.0048 (17)	−0.0008 (18)
C51	0.034 (2)	0.051 (3)	0.022 (2)	0.000 (2)	0.0056 (18)	0.001 (2)
C52	0.039 (2)	0.051 (3)	0.027 (3)	0.009 (2)	0.004 (2)	0.013 (2)
C53	0.042 (2)	0.033 (2)	0.033 (3)	0.0073 (19)	0.006 (2)	0.0130 (19)
C54	0.0247 (19)	0.027 (2)	0.028 (2)	0.0035 (16)	0.0063 (17)	0.0041 (17)
C55	0.027 (2)	0.0190 (19)	0.036 (2)	0.0009 (16)	0.0077 (18)	0.0044 (17)
C56	0.051 (3)	0.020 (2)	0.044 (3)	0.0065 (19)	0.008 (2)	0.0079 (19)
C57	0.053 (3)	0.019 (2)	0.044 (3)	0.0058 (19)	0.012 (2)	−0.0057 (19)
C58	0.041 (2)	0.028 (2)	0.032 (3)	0.0017 (18)	0.007 (2)	−0.0066 (18)
C59	0.031 (2)	0.024 (2)	0.028 (2)	−0.0018 (16)	0.0105 (17)	−0.0005 (17)
N5	0.0243 (16)	0.0177 (15)	0.0231 (18)	0.0022 (12)	0.0063 (13)	−0.0008 (13)
O4	0.0152 (12)	0.0297 (14)	0.0251 (15)	0.0003 (10)	0.0045 (11)	−0.0017 (11)
C60	0.0194 (17)	0.0161 (18)	0.034 (2)	−0.0007 (14)	0.0106 (16)	−0.0034 (16)
C61	0.0213 (18)	0.022 (2)	0.033 (2)	0.0044 (15)	0.0059 (17)	0.0019 (17)
C62	0.0186 (18)	0.028 (2)	0.045 (3)	0.0024 (16)	0.0122 (18)	−0.0024 (19)
C63	0.032 (2)	0.037 (2)	0.037 (3)	0.0011 (18)	0.022 (2)	0.003 (2)
C64	0.0243 (19)	0.030 (2)	0.032 (2)	−0.0013 (16)	0.0115 (17)	0.0013 (18)
C65	0.0182 (17)	0.0153 (18)	0.031 (2)	0.0000 (14)	0.0061 (16)	0.0016 (15)
C66	0.0237 (18)	0.0206 (19)	0.022 (2)	−0.0025 (15)	0.0043 (16)	−0.0005 (15)
N6	0.0169 (14)	0.0203 (16)	0.0217 (17)	0.0021 (12)	0.0048 (13)	0.0016 (13)
C67	0.0180 (17)	0.025 (2)	0.019 (2)	0.0055 (14)	0.0063 (15)	0.0042 (15)
C68	0.0221 (18)	0.0197 (18)	0.019 (2)	0.0025 (14)	0.0076 (15)	−0.0015 (15)
C69	0.0181 (17)	0.026 (2)	0.020 (2)	−0.0009 (15)	0.0081 (15)	−0.0013 (15)
C70	0.0212 (18)	0.028 (2)	0.022 (2)	0.0083 (15)	0.0075 (15)	0.0006 (16)
C71	0.0274 (19)	0.0192 (19)	0.027 (2)	0.0042 (15)	0.0086 (17)	0.0020 (16)
C72	0.0223 (18)	0.0226 (19)	0.027 (2)	0.0025 (15)	0.0086 (16)	−0.0013 (16)
O5	0.0200 (13)	0.0282 (15)	0.0367 (17)	−0.0006 (11)	0.0045 (12)	0.0001 (12)
C73	0.0225 (19)	0.032 (2)	0.039 (3)	−0.0049 (17)	0.0066 (18)	−0.0071 (19)
O6	0.0207 (13)	0.0293 (15)	0.0337 (16)	0.0078 (11)	0.0034 (12)	0.0008 (12)
C74	0.036 (2)	0.032 (2)	0.042 (3)	0.0153 (19)	0.003 (2)	0.007 (2)
C75	0.045 (3)	0.084 (4)	0.067 (4)	−0.006 (3)	0.007 (3)	−0.024 (3)
Cl1	0.0607 (9)	0.0871 (11)	0.0785 (11)	0.0087 (8)	−0.0081 (8)	−0.0318 (9)
Cl2	0.0603 (10)	0.1180 (16)	0.161 (2)	0.0132 (10)	−0.0006 (12)	−0.0604 (15)
C76	0.044 (3)	0.052 (3)	0.047 (3)	−0.004 (2)	0.014 (2)	−0.006 (2)
Cl3	0.0760 (10)	0.0577 (9)	0.0758 (10)	−0.0187 (7)	0.0347 (8)	−0.0137 (7)
Cl4	0.0976 (12)	0.0687 (10)	0.0833 (12)	−0.0258 (9)	0.0503 (10)	−0.0310 (9)
C77	0.034 (2)	0.035 (2)	0.039 (3)	−0.0043 (19)	0.010 (2)	−0.001 (2)
Cl5	0.0349 (5)	0.0344 (6)	0.0472 (7)	−0.0012 (4)	0.0095 (5)	−0.0049 (5)
Cl6	0.0409 (6)	0.0405 (6)	0.0466 (7)	0.0017 (5)	0.0120 (5)	−0.0076 (5)
C78	0.051 (3)	0.056 (3)	0.049 (3)	−0.012 (2)	0.014 (3)	0.004 (3)
Cl8A	0.066 (8)	0.046 (4)	0.027 (5)	−0.035 (4)	0.015 (4)	−0.013 (4)
Cl8B	0.239 (7)	0.0434 (13)	0.051 (3)	−0.030 (2)	0.062 (4)	−0.0124 (12)
Cl7	0.1229 (13)	0.0366 (7)	0.0646 (10)	−0.0190 (8)	0.0417 (9)	−0.0050 (6)

### Geometric parameters (Å, º)

**Table:**

Ir1—N1	2.028 (3)	Ir2—N6	2.146 (3)
Ir1—C11	1.998 (4)	C38—C39	1.398 (5)
Ir1—C12	1.996 (4)	C38—C43	1.413 (5)
Ir1—N2	2.036 (3)	C39—C40	1.390 (5)
Ir1—O1	2.147 (2)	C40—C41	1.380 (6)
Ir1—N3	2.149 (3)	C41—C42	1.380 (6)
N1—C1	1.347 (5)	C42—C43	1.402 (5)
N1—C5	1.357 (5)	C43—C44	1.462 (5)
C1—C2	1.374 (5)	C44—C45	1.398 (5)
C2—C3	1.386 (6)	C44—N4	1.360 (5)
C3—C4	1.370 (6)	C45—C46	1.363 (6)
C4—C5	1.392 (5)	C46—C47	1.381 (6)
C5—C6	1.464 (5)	C47—C48	1.372 (5)
C6—C7	1.396 (5)	C48—N4	1.341 (5)
C6—C11	1.419 (5)	C49—C50	1.395 (5)
C7—C8	1.387 (6)	C49—C54	1.402 (5)
C8—C9	1.376 (6)	C50—C51	1.389 (6)
C9—C10	1.389 (5)	C51—C52	1.379 (6)
C10—C11	1.395 (5)	C52—C53	1.381 (6)
C12—C13	1.409 (5)	C53—C54	1.400 (5)
C12—C17	1.413 (5)	C54—C55	1.468 (6)
C13—C14	1.379 (5)	C55—C56	1.389 (5)
C14—C15	1.387 (6)	C55—N5	1.358 (5)
C15—C16	1.383 (6)	C56—C57	1.378 (6)
C16—C17	1.400 (5)	C57—C58	1.378 (6)
C17—C18	1.458 (5)	C58—C59	1.374 (5)
C18—C19	1.388 (5)	C59—N5	1.345 (5)
C18—N2	1.369 (5)	O4—C60	1.303 (4)
C19—C20	1.372 (6)	C60—C61	1.416 (5)
C20—C21	1.379 (6)	C60—C65	1.423 (5)
C21—C22	1.380 (5)	C61—C62	1.368 (5)
C22—N2	1.348 (5)	C62—C63	1.393 (6)
O1—C23	1.304 (4)	C63—C64	1.379 (5)
C23—C24	1.421 (5)	C64—C65	1.401 (5)
C23—C28	1.416 (5)	C65—C66	1.452 (5)
C24—C25	1.361 (5)	C66—N6	1.283 (4)
C25—C26	1.396 (6)	N6—C67	1.447 (4)
C26—C27	1.371 (5)	C67—C68	1.388 (5)
C27—C28	1.412 (5)	C67—C72	1.382 (5)
C28—C29	1.442 (5)	C68—C69	1.379 (5)
C29—N3	1.285 (5)	C69—C70	1.403 (5)
N3—C30	1.439 (4)	C69—O5	1.371 (4)
C30—C31	1.376 (5)	C70—C71	1.374 (5)
C30—C35	1.399 (5)	C70—O6	1.375 (4)
C31—C32	1.405 (5)	C71—C72	1.394 (5)
C32—C33	1.371 (5)	O5—C73	1.425 (4)
C33—C34	1.409 (5)	O6—C74	1.423 (5)
C33—O2	1.378 (4)	C75—Cl1	1.757 (6)
C34—C35	1.378 (5)	C75—Cl2	1.727 (6)
C34—O3	1.364 (4)	C76—Cl3	1.761 (5)
O2—C36	1.430 (5)	C76—Cl4	1.748 (5)
O3—C37	1.429 (5)	C77—Cl5	1.770 (4)
Ir2—C38	2.000 (4)	C77—Cl6	1.766 (4)
Ir2—N4	2.030 (3)	C78—Cl8A	1.748 (5)
Ir2—C49	2.010 (4)	C78—Cl8B	1.784 (4)
Ir2—N5	2.037 (3)	C78—Cl7	1.727 (5)
Ir2—O4	2.151 (2)		
			
C11—Ir1—N1	80.64 (14)	C49—Ir2—N4	96.13 (14)
C12—Ir1—N1	94.36 (14)	N5—Ir2—C38	97.04 (14)
C12—Ir1—C11	89.53 (14)	N5—Ir2—N4	176.06 (12)
N2—Ir1—N1	174.57 (12)	N5—Ir2—C49	80.52 (14)
N2—Ir1—C11	97.33 (13)	O4—Ir2—C38	174.17 (13)
N2—Ir1—C12	80.55 (14)	O4—Ir2—N4	95.39 (11)
O1—Ir1—N1	94.86 (11)	O4—Ir2—C49	89.50 (12)
O1—Ir1—C11	175.03 (13)	O4—Ir2—N5	86.69 (11)
O1—Ir1—C12	88.70 (12)	N6—Ir2—C38	97.25 (13)
O1—Ir1—N2	86.97 (10)	N6—Ir2—N4	86.08 (12)
N3—Ir1—N1	86.66 (11)	N6—Ir2—C49	175.75 (12)
N3—Ir1—C11	96.53 (13)	N6—Ir2—N5	97.40 (12)
N3—Ir1—C12	173.93 (12)	N6—Ir2—O4	86.67 (10)
N3—Ir1—N2	98.60 (12)	C39—C38—Ir2	128.3 (3)
N3—Ir1—O1	85.26 (10)	C43—C38—Ir2	114.2 (3)
C1—N1—Ir1	123.0 (3)	C43—C38—C39	117.4 (3)
C5—N1—Ir1	116.7 (2)	C40—C39—C38	121.0 (4)
C5—N1—C1	120.3 (3)	C41—C40—C39	121.2 (4)
C2—C1—N1	121.5 (4)	C42—C41—C40	119.2 (4)
C3—C2—C1	118.9 (4)	C43—C42—C41	120.6 (4)
C4—C3—C2	119.8 (4)	C42—C43—C38	120.7 (4)
C5—C4—C3	119.8 (4)	C44—C43—C38	115.2 (3)
C4—C5—N1	119.8 (4)	C44—C43—C42	124.1 (4)
C6—C5—N1	113.4 (3)	C45—C44—C43	127.2 (4)
C6—C5—C4	126.8 (4)	N4—C44—C43	113.6 (3)
C7—C6—C5	123.8 (4)	N4—C44—C45	119.2 (4)
C11—C6—C5	115.1 (3)	C46—C45—C44	120.5 (4)
C11—C6—C7	121.1 (4)	C47—C46—C45	119.3 (4)
C8—C7—C6	119.8 (4)	C48—C47—C46	118.9 (4)
C9—C8—C7	119.9 (4)	N4—C48—C47	122.1 (4)
C10—C9—C8	120.7 (4)	C44—N4—Ir2	116.3 (3)
C11—C10—C9	121.4 (4)	C48—N4—Ir2	123.8 (3)
C6—C11—Ir1	114.2 (3)	C48—N4—C44	119.9 (3)
C10—C11—Ir1	128.7 (3)	C50—C49—Ir2	128.5 (3)
C10—C11—C6	117.1 (3)	C54—C49—Ir2	114.5 (3)
C13—C12—Ir1	128.6 (3)	C54—C49—C50	117.0 (4)
C17—C12—Ir1	114.7 (3)	C51—C50—C49	121.2 (4)
C17—C12—C13	116.6 (3)	C52—C51—C50	120.9 (4)
C14—C13—C12	121.4 (4)	C53—C52—C51	119.6 (4)
C15—C14—C13	121.1 (4)	C54—C53—C52	119.5 (4)
C16—C15—C14	119.4 (4)	C53—C54—C49	121.8 (4)
C17—C16—C15	119.9 (4)	C55—C54—C49	115.1 (3)
C16—C17—C12	121.6 (4)	C55—C54—C53	123.1 (4)
C18—C17—C12	114.9 (3)	C56—C55—C54	125.9 (4)
C18—C17—C16	123.5 (4)	N5—C55—C54	114.0 (3)
C19—C18—C17	126.8 (4)	N5—C55—C56	120.1 (4)
N2—C18—C17	113.8 (3)	C57—C56—C55	120.0 (4)
N2—C18—C19	119.4 (4)	C58—C57—C56	119.5 (4)
C20—C19—C18	121.0 (4)	C59—C58—C57	118.5 (4)
C21—C20—C19	119.1 (4)	N5—C59—C58	122.6 (4)
C22—C21—C20	118.6 (4)	C55—N5—Ir2	115.9 (3)
N2—C22—C21	122.6 (4)	C59—N5—Ir2	124.8 (3)
C18—N2—Ir1	115.8 (2)	C59—N5—C55	119.3 (3)
C22—N2—Ir1	125.0 (3)	C60—O4—Ir2	124.1 (2)
C22—N2—C18	119.2 (3)	C61—C60—O4	118.4 (3)
C23—O1—Ir1	121.7 (2)	C65—C60—O4	125.0 (3)
C24—C23—O1	119.1 (3)	C65—C60—C61	116.6 (3)
C28—C23—O1	124.4 (3)	C62—C61—C60	122.2 (4)
C28—C23—C24	116.4 (3)	C63—C62—C61	121.4 (4)
C25—C24—C23	122.2 (4)	C64—C63—C62	117.7 (4)
C26—C25—C24	121.0 (4)	C65—C64—C63	122.7 (4)
C27—C26—C25	118.7 (4)	C64—C65—C60	119.5 (3)
C28—C27—C26	121.5 (4)	C66—C65—C60	123.7 (3)
C27—C28—C23	120.1 (3)	C66—C65—C64	116.8 (3)
C29—C28—C23	123.3 (3)	N6—C66—C65	127.7 (3)
C29—C28—C27	116.5 (3)	C66—N6—Ir2	124.7 (2)
N3—C29—C28	126.7 (4)	C67—N6—Ir2	119.6 (2)
C29—N3—Ir1	124.0 (2)	C67—N6—C66	115.1 (3)
C30—N3—Ir1	119.8 (2)	C68—C67—N6	119.1 (3)
C30—N3—C29	115.6 (3)	C72—C67—N6	120.4 (3)
C31—C30—N3	120.4 (3)	C72—C67—C68	120.5 (3)
C35—C30—N3	119.2 (3)	C69—C68—C67	119.8 (3)
C35—C30—C31	120.4 (3)	C70—C69—C68	120.0 (3)
C32—C31—C30	119.5 (4)	O5—C69—C68	124.2 (3)
C33—C32—C31	120.4 (4)	O5—C69—C70	115.8 (3)
C34—C33—C32	119.9 (3)	C71—C70—C69	119.7 (3)
O2—C33—C32	124.4 (4)	O6—C70—C69	114.9 (3)
O2—C33—C34	115.7 (3)	O6—C70—C71	125.4 (3)
C35—C34—C33	119.8 (3)	C72—C71—C70	120.5 (3)
O3—C34—C33	115.9 (3)	C71—C72—C67	119.5 (3)
O3—C34—C35	124.3 (3)	C73—O5—C69	116.8 (3)
C34—C35—C30	119.9 (3)	C74—O6—C70	116.5 (3)
C36—O2—C33	116.8 (3)	Cl2—C75—Cl1	111.8 (3)
C37—O3—C34	116.4 (3)	Cl4—C76—Cl3	112.5 (3)
N4—Ir2—C38	80.63 (14)	Cl6—C77—Cl5	111.7 (2)
C49—Ir2—C38	86.71 (14)		

### Hydrogen-bond geometry (Å, º)

Cg1 and Cg2 are the centroids of the N1/C1–C5 and N4/C44–C48 rings, respectively.

**Table:**

*D*—H···*A*	*D*—H	H···*A*	*D*···*A*	*D*—H···*A*
C1—H1···O1	0.95 (1)	2.51 (1)	3.112 (5)	121 (1)
C13—H13···*Cg*1	0.95 (1)	3.06 (4)	3.83 (4)	139 (1)
C74—H74*a*···*Cg*1	0.98 (1)	3.40 (17)	4.12 (5)	132 (2)
C48—H48···O4	0.95 (1)	2.56 (1)	3.155 (5)	121 (1)
C36—H36*c*···*Cg*2	0.98 (1)	3.47 (19)	4.18 (5)	131 (2)
C50—H50···*Cg*2	0.95 (1)	3.22 (4)	3.98 (4)	138 (1)
C29—H29···O2^i^	0.95 (1)	2.60 (1)	3.152 (5)	118 (1)
C29—H29···O3^i^	0.95 (1)	2.52 (1)	3.463 (5)	172 (1)
C58—H58···O6^ii^	0.95 (1)	2.39 (1)	3.330 (5)	170 (1)
C75—H75A···O4^iii^	0.99 (1)	2.35 (1)	3.310 (7)	165 (1)
C77—H77A···O1^iv^	0.99 (1)	2.19 (1)	3.172 (7)	172 (1)

Symmetry codes: (i) −*x*+1, −*y*+1, −*z*; (ii) −*x*+1, −*y*+2, −*z*+1; (iii) *x*+1, *y*, *z*; (iv) *x*−1, *y*, *z*.
